# Susceptibility of Commensal *E. coli* Isolated from Conventional, Antibiotic-Free, and Organic Meat Chickens on Farms and at Slaughter toward Antimicrobials with Public Health Relevance

**DOI:** 10.3390/antibiotics10111321

**Published:** 2021-10-29

**Authors:** Laura Musa, Patrizia Casagrande Proietti, Maria Luisa Marenzoni, Valentina Stefanetti, Tana Shtylla Kika, Francesca Blasi, Chiara Francesca Magistrali, Valeria Toppi, David Ranucci, Raffaella Branciari, Maria Pia Franciosini

**Affiliations:** 1 Department of Veterinary Medicine, Via S. Costanzo 4, 06126 Perugia, Italy; laura.musa@studenti.unipg.it (L.M.); marialuisa.marenzoni@unipg.it (M.L.M.); valentina.stefanetti@unipg.it (V.S.); toppivaleria@gmail.com (V.T.); david.ranucci@unipg.it (D.R.); raffaella.branciari@unipg.it (R.B.); maria.franciosini@unipg.it (M.P.F.); 2 Department of Veterinary Public Health, Faculty of Veterinary Medicine, Agricultural University of Tirana, Koder Kamez, 1029 Tirana, Albania; tana.shtylla@ubt.edu.al; 3 Istituto Zooprofilattico Sperimentale dell’Umbria e delle Marche ‘Togo Rosati’, 06124 Perugia, Italy; f.blasi@izsum.it (F.B.); c.magistrali@izsum.it (C.F.M.)

**Keywords:** broiler chicken, antimicrobial susceptibility, microdilution test, commensal *E. coli*, organic system, antibiotic-free system, conventional system

## Abstract

The spread of resistant bacteria from livestock to the food industry promoted an increase of alternative poultry production systems, such as organic and antibiotic-free ones, based on the lack of antimicrobial use, except in cases in which welfare is compromised. We aimed to investigate the antibiotic susceptibility of commensal *Escherichia coli* isolated from organic, antibiotic-free, and conventional broiler farms and slaughterhouses toward several antimicrobials critically important for human health. To assess antimicrobial susceptibility, all *E. coli* isolates and extended spectrum beta-lactamase (ESBL) *E. coli* were analysed by the microdilution method. The prevalence of tigecycline, azithromycin and gentamicin *E. coli*-resistant strains was highest in organic samplings. Conversely, the lowest prevalence of resistant *E. coli* strains was observed for cefotaxime, ceftazidime and ciprofloxacin in organic systems, representing a significant protective factor compared to conventional systems. All *E. coli* strains were colistin-susceptible. Contamination of the external environment by drug-resistant bacteria could play a role in the presence of resistant strains detected in organic systems. Of interest is the highest prevalence of cephalosporin resistance of *E. coli* in conventional samplings, since they are not permitted in poultry. Our results suggest that monitoring of antibiotic resistance of the production chain may be helpful to detect “risks” inherent to different rearing systems.

## 1. Introduction

Over the years, the rapidly increasing demand for livestock products by the human population has led to the development of intensive production of food animals, such as cattle, poultry and pigs [1], and the increase of antimicrobial resistance, due to the uncontrolled use of antimicrobials [2,3] for the prevention and treatment of infections, promotion of growth and improvement in production [4].

Furthermore, waste from hospitals and livestock producers containing antimicrobial residues have facilitated the selection of resistomes in the environment with potential diffusion to animals and humans [5]. In this context, *Escherichia coli,* along with *Salmonella* and *Campylobacter* [6,7], is one of the bacteria most responsible for resistant gene co-circulation among the environment, animals and humans. In addition to resistance against classical molecules that have been overused in the zootechnical sector, there has been an increase in the prevalence of resistance toward antimicrobials prevalently used in human medicine, such as extended-spectrum cephalosporins (ESC), including the third- and fourth-generation cephalosporins [8], neomycin, apramycin and tigecycline [9,10]. Tigecycline with colistin is considered to be a last resort defence against infections due to carbapenem-resistant *Enterobacteriaceae* and multidrug resistant (MDR) Gram-negative bacteria [11,12]. Decreased susceptibility to colistin and tigecycline are justified by encoded intrinsic resistance and the presence of mobile colistin and tigecycline resistance genes [13,14]. A transferable plasmid-derived colistin resistance gene *mcr-1* was found to be responsible for resistance occurrence worldwide [15,16]. It should be considered that colistin has been widely administered for the prevention, treatment, metaphylaxis and growth promotion in veterinary medicine for years [17,18]. Among food-producing animals, several studies have reported a notable prevalence of colistin resistance in poultry [19,20,21]. It should be stressed that there has been an alarming increase of bacteria resistant to third-generation cephalosporins, which reinforces the suspicion of possible “non-official” use in chicks at hatch [22,23]. Actually, it is known that the use of cephalosporins in poultry and other species was prohibited by the Food and Drug Administration [24], since they could be responsible for triggering resistance to these classes of antimicrobials in humans. During the last decade, a progressive increase in ESBL *E. coli* associated with a multi-resistance profile has also been documented on chicken farms [25]. This, in turn, has caused concern for public health, as poultry meat is widely consumed, both for its nutritional characteristics and the economic benefits related to low costs [26]. Moreover, the diffusion of ESBL could result in unsuccessful therapeutic treatment in human infections and also require the use of “last resort antibiotics” (e.g., carbapenems) causing an increased resistance to these antibiotic classes [27,28]. In this scenario, poultry producers have turned to alternative production systems, such as organic (O) and antibiotic-free (AF) farming, based on the lack of antimicrobial use, unless animal welfare is at risk [29]. In our work, we aimed to investigate the antibiotic susceptibility of commensal *E. coli*, especially towards antimicrobials used in human therapy, isolated in organic, antibiotic-free and conventional (C) broilers on farms and at slaughter. The diffusion of ESBL *E. coli* was also assayed.

## 2. Results

**Figure 1:**
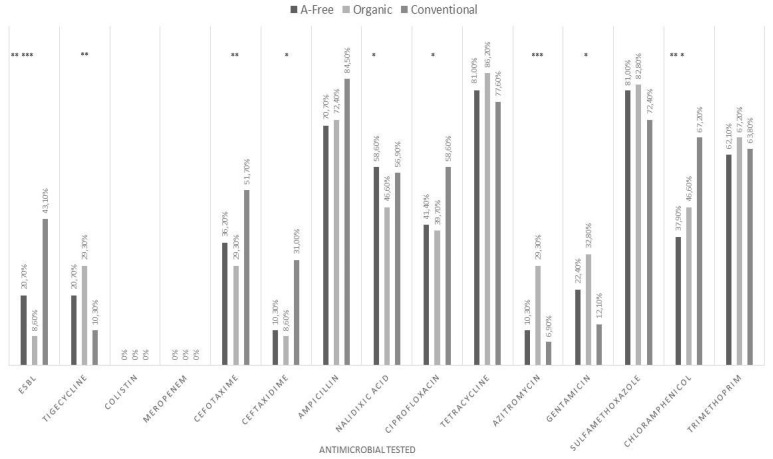
Prevalence of antimicrobial-resistant *E. coli* strains in conventional, organic and antibiotic-free systems. * *p* ≤ 0.05; ** *p* ≤ 0.01; *** *p* ≤ 0.001.

With respect to antimicrobial susceptibility, O and AF samplings showed higher prevalence of *E. coli*-resistant strains to tigecycline than C (29.3% in O, 20.7% in AF, and 10.3% in C) (Figure 1) with an odds ratio (OR) of 3.59 (*p* = 0.01) for O (Table 1). All *E. coli* strains isolated from O, AF and C had minimal inhibitory concentration (MIC) values for tigecycline within 1–4 μg/mL (Table S1). The O samplings showed the highest prevalence of *E. coli*-resistant strains for azithromycin (29.3% vs. 10.3% in AF and 6.9% in C) with OR = 4.39 (*p* = 0.001), and for gentamicin (32.8% vs. 22.4% in AF and 12.1% in C) with OR = 2.34 (*p* = 0.02) when compared to the C systems (Figure 1 and Table 1). The MIC values for the most azithromycin-resistant *E. coli* strains isolated from O samplings were greater than 64 μg/mL (Table S1). The O samplings exhibited the lowest prevalence of resistance of *E. coli* to cefotaxime and ceftazidime-resistant strains. The prevalence rates of *E. coli* cefotaxime-resistant strains were 29.3% in O, 36.2% in AF and 51.7% in C with OR = 0.39 (*p* = 0.01) and the prevalence of ceftazidime-resistant strains was 8.6% in O, 10.3% in AF and 31% in C with an OR of 0.36 (*p* = 0.02) (Figure 1 and Table 1).

The lowest prevalence for *E. coli* ciprofloxacin-resistant strains was detected in O samplings (39.7% vs. 58.6% in C and 41.4% in AF with OR = 0.46, (*p* = 0.04) (Figure 1 and Table 1). The highest prevalence of *E. coli* chloramphenicol-resistant strains was found in C samplings (67.2% in C, 46.6% in O and 37.9% in AF) (Figure 1). The prevalence of nalidixic acid-resistant *E. coli* in the AF, C and O systems was 58.6%, 56.9% and 46.6%, respectively (Figure 1). The majority of the resistant strains displayed MIC values ≥ 128 μg/mL for nalidixic acid (Table S1). All *E. coli* strains isolated from the O and C samplings were susceptible to meropenem (Table S1)**.** We isolated 10.3% of susceptible, increased exposure [30] *E. coli* to meropenem from AF samplings at slaughter with 4 μg/mL MIC values. All *E. coli* strains were colistin-susceptible (MIC range, 0.5–1 μg/mL) (Table S1). The lowest prevalence of ESBL *E. coli* was found in the O and AF samplings (8.6% in O, 20.7% in AF and 43.1% in C) (Figure 1, with an OR of 0.13 (*p* < 0.001) and OR = 0.33 (*p* = 0.009), respectively (Figure 1 and Table 1). Farm, when compared to slaughterhouse, resulted in a protective factor for the presence of ESBL *E. coli* strains (OR = 2.72; *p* = 0.01) (Table 1).

No statistical differences were found in the multi-resistance among the *E. coli* isolates from the three systems. Most of the *E. coli* strains were resistant to five (AF, 22.1%; O, 30.6%; and C, 28.9%) and six antimicrobials (AF, 23.8%; O, 23.8%; and C, 17.0%) (Table S2).

## 3. Discussion

*Escherichia coli* is a normal constituent of the intestinal microbial flora of poultry, although it is also a potential pathogen associated with serious diseases, such as colibacillosis. Moreover, it can also represent a potential source of resistant genes transferable to humans, posing a public health threat [31]. In this context, we aimed to investigate if the different typology of farming can influence the antimicrobial susceptibility of *E. coli* isolated on farms and at slaughter. Our study revealed that the organic and antibiotic-free samplings showed the highest prevalence of *E. coli*-resistant strains to tigecycline. The conventional system resulted in a significant protective factor for the presence of the tigecycline-resistant strain of *E. coli* when compared to organic and antibiotic-free management. Tigecycline is a broad-spectrum glycylcycline synthesised to overcome tetracycline resistance [32]. Its use has indeed developed in response to increasing resistance of *Staphylococcus aureus*, *Acinetobacter baumannii*, and *E. coli* to a majority of antimicrobials [33]. In order to maintain tigecycline efficacy, the European Commission (EC) requested that the European Medicine Agency (EMA) provide scientific advice on the impact of its use in animals on human health and recommend the restricted use of glycylcyclines in veterinary medicine. Tigecycline has not really been used on conventional poultry farms and the high prevalence of *E. coli*-resistant strains found in organic systems could likely be dependent on the resistant strains present in the external environment. Recently, Sun et al. [34] demonstrated a significant presence of tigecycline-resistant *E. coli* strains from raw meat bearing the *tet* (X) gene. It should also be highlighted that in our investigation, all *E. coli* strains were susceptible to colistin differently than what was observed in other studies [35,36,37]. However, it should be specified that colistin, after being administrated for a long time as a prophylactic and therapeutic agent, is currently in off-label use in Italy and it was banned as a growth promoter [35]. The large use in poultry has been favoured by the lack of withdrawal time for eggs and meat [38,39]. Moreover, colistin, though considered efficient against human *Enterobacteriaceae* infections, was replaced because of its systemic toxicity [40]. Recently, the emergence of the MDR (multidrug resistant ) bacteria, responsible for severe infections and deaths in humans, have led to addressing the reconsideration of the colistin as a last-resort antibiotic against these “superbug” bacteria [12]. Additionally of interest are the results related to the prevalence of azithromycin and gentamicin-resistant *E. coli* strains, which were found to be higher in organic samples than in antibiotic-free and conventional ones. It should be considered that azithromycin has not been used in poultry and the results observed in organic samples could also be justified by external contamination likely due to wildlife [41,42]. Recently, azithromycin became the first choice drug for the treatment of enterotoxigenic *E. coli* (ETEC) infections in human medicine since a progressive resistance has been described for nalidixic acid, ampicillin, sulphonamides and tetracycline [43]. Gentamicin has been used against poultry diseases, such as respiratory diseases and necrotic enteritis [44,45]. At present, this medication is largely administered in industrial poultry production due to poor intestinal absorption and the lack of residues in edible chicken tissues [39,46]. The prevalence of ceftazidime and cefotaxime *E. coli*-resistant strains were highest in the conventional samplings. Thus, the organic system represented a significant protective factor for the presence of cefotaxime, ceftazidime and ciprofloxacin-resistant strain *E. coli* when compared to C management. Several *E. coli* strains have acquired the capability to inactivate a large number of β-lactam antibiotics, including penicillin and extended-spectrum cephalosporins, such as third- and fourth-generation cephalosporins [47]. Furthermore, in the last years ESBL/AmpC *E. coli* has been frequently found as a contaminant in broiler meat [48]. It should be mentioned that the increased use of amoxicillin and ampicillin in poultry therapy might have promoted the rise of ESBL *E. coli.* In particular, these antimicrobials have been used against enteric disorders associated with an overgrowth of *Clostridium perfringens* [49,50] following the ban of the use of antibiotics as feed growth promoters in industrial chickens [51]. Our results showed that antibiotic-free and organic systems, that had not used antimicrobials, were protective factors for ESBL presence if compared to conventional management.

Furthermore, we observed the highest prevalence of chloramphenicol-resistant *E. coli* strains in conventional samples. The use of chloramphenicol in livestock has been banned in Europe since 1997 [29]. In our work, the resistance to chloramphenicol described in the conventional samplings could be explained by the possible effects of repeated antibiotic treatments with amoxicillin, as described for broilers [52]. Recently, Burow et al. [53] have experimentally demonstrated an increase in resistance towards several antimicrobials (tetracycline, ciprofloxacin, nalidixic acid, gentamicin) after oral administration of amoxicillin, which is commonly used in poultry practice. Finally, ESBL *E. coli* strains were higher at slaughter than in the farm. A cross-contamination mechanism can occur during scalding and/or evisceration and dressing, although contamination with ESBL-producing *Enterobacteriaceae* may be due to workers responsible for the contamination of live animals, carcasses or organs, as occurs in pigs [54]. With respect to the detection of MDR strains, five and six patterns of multi-resistance were found, although no statistical differences in prevalence were seen among the different rearing systems. Previous works have demonstrated that meat products from conventionally raised poultry exhibited MDR bacteria more frequently than meat products from antibiotic-free or organic chickens [55,56], although some exceptions [57,58] have been reported.

## 4. Materials and Methods

### 4.1. Sampling

A total of 174 strains were collected from samplings performed on conventional (C), organic (O) and antibiotic-free (AF) chicken farms (cloacal swabs and environmental samples) and at slaughter (caecal contents and skin samples). In particular, 58 strains of *E. coli* were collected for each typology of farming: in C (25 and 33 on farm and in slaughterhouse, respectively), in O (31 and 27 on farm and in slaughterhouse, respectively) and in AF (25 and 33 on farm and in slaughterhouse, respectively).

### 4.2. Isolation and Identification of E. coli

All samples were placed in a pre-enrichment medium consisting of buffered peptone water (BPW) at a ratio of 1:10 and then incubated at 37 °C for 18–24 h in aerobiosis, after which 0.1 mL from each diluted sample was plated on MacConkey agar with an added low concentration (1 mg/L) of cefotaxime (Thermo Fisher Scientific, Milan, Italy). The plates were incubated for 24 h at 37 °C under aerobic conditions. All colonies with typical *E. coli* morphology were selected and confirmed by biochemical tests [59].

### 4.3. Antibiotic Susceptibility Testing and ESBL E. coli Detection

To assess antimicrobial susceptibility, all *E. coli* isolates were analysed by the broth microdilution method. Pure cultures were suspended in 4 mL of 0.90% sterile saline solution (final concentration: 5 × 10^7^ CFU/mL CFU/mL^−1^), equivalent to a 0.5 McFarland turbidity level (Vitek, bioMérieux Inc., Durham, United States). Ten microlitres of bacterial suspension was transferred to 11 mL of cation-adjusted Mueller Hinton broth (Thermo Fisher Scientific, Milan, Italy) and 50 μL of bacterial suspension was dispensed into each well of Euvsec microtitre plates (Thermo Fisher Scientific, Milan, Italy) with scalar concentrations of the following antibiotics: sulfamethoxazole (SMX) (8–1024 μg/mL), trimethoprim (TMP) (0.25–32 μg/mL), ciprofloxacin (CIP) (0.03–8 μg/mL), tetracycline (TET) (2–64 μg/mL), meropenem (MERO) (0.06–16μg/mL), azithromycin (AZI) (2–64 μg/mL), nalidixic acid (NAL) (4–128 μg/mL), cefotaxime (FOT) (0.25–1 μg/mL), chloramphenicol (CHL) (8–128 μg/mL), tigecycline (TGC) (0.25–8 μg/mL), ceftazidime (TAZ) (2–8 μg/mL), colistin (COL) (1–16 μg/mL), ampicillin (AMP) (1–64 μg/mL), and gentamicin (GEN) ( 0.5–32 μg/mL). After inoculation, the plates were incubated for 24 h at 37 °C under aerobic conditions. Susceptibility to colistin was evaluated using the FRCOL Plates (0.12–128 μg/mL) (Thermo Fisher Scientific, Milan, Italy). For all *E. coli*, ESBL production was confirmed by the double-disc synergy test (DDST) [59] and the microdilution method using Sensititre™ ESBL plates (Thermo Fisher Scientific, Milan, Italy). *E. coli* ATCC 25922 was used as the quality control strain. The results were evaluated according to the breakpoints established by the European Committee on Antimicrobial Susceptibility Testing (EUCAST) [60], with the exception of sulfamethoxazole, tetracycline, azithromycin and nalidixic acid, for which breakpoints published by the Clinical and Laboratory Standard Institute (CLSI) were applied [61].

### 4.4. Statistical Analysis

Logistic regression was used to weigh the overall effect of the types of rearing systems (C, AF and O) or the effect of the sampling on farm or at slaughterhouse level on antibiotic resistance. These variables were examined separately for their association with antibiotic-resistant *E. coli* strains and the presence of EBSL *E. coli*. Variables scoring *p* ≤ 0.20 in an early univariate analysis were included in the regression model. Non-significant variables were manually removed from the model. ORs and corresponding 95% confidence intervals (CIs) were obtained by means of logistic regression. Data were analysed by commercial software R, version 2.8.1 (R, Development Core Team 2007). A value of *p* ≤ 0.05 was considered statistically significant for the analysis.

## 5. Conclusions

Our research evidenced that rearing systems can influence the antimicrobial susceptibility of *E. coli*, as seen in the organic system for resistance to some antibiotics, such as tigecycline and azithromycin, used in human therapy. The organic system represents a protective factor if compared to conventional system for the lowest prevalence of *E. coli*-resistant strains to cefotaxime and ceftazidime. Our data evidenced that the environment can represent an important source of resistant bacteria for organic farming. Wild animals [62], insects [63], wastewater, and zootechnical waste [64] used as fertilizers are described to contaminate the environment in relation to the geographical area. Based on our data it is not still possible to be conclusive in individuating the “ideal farming” in relation to antibiotic-resistance relevance. There are also numerous components influencing the choice of the system, such as the geographical area and the economic factors. Farming is generally a protective factor if compared to slaughterhouse, in relation to the prevalence of ESBL *E. coli* strains. Thus, particular attention must be paid to the hygiene of the slaughtering process to avoid cross-contamination that can influence the best practices adopted at the farm level to reduce the prevalence of ESBL and antibiotic resistance bacteria in poultry meat. Continuous epidemiological monitoring of the antibiotic resistance of the production chain could be helpful to notice situations of “risk” inherent to different types of farming systems in order to apply corrective factors to various categories of production (control of the breeders) and different rearing systems (i.e., control of wildlife and environment for organic farming), as well as at slaughter (carcasses and environmental contamination), in order to optimise final production and guarantee food safety for consumers.

## Figures and Tables

**Table 1:** Multivariate logistic regression analysis for ESBL *E. coli*, MDR *E. coli* strain prevalence and *E. coli* antimicrobial susceptibility (only significant ORs, and their 95% confidence interval, are reported in the table).

Outcome	Explanatory Variable	Level		OR *	95 % Confidence Interval		*p*-Value
					Lower Limit	Upper Limit	
ESBL strains	Type of rearing	Conventional	ref ^a^	-	-	-	
		Organic		0.13	0.04	0.37	<0.001
		AF		0.33	0.14	0.76	0.009
	Site of sampling	Farm	ref	-	-	-	
		Slaughterhouse		2.72	1.23	6.02	0.01
MDR strains	Type of rearing	Conventional		-	-	-	
		Organic		-	-	-	0.17
		AF		-	-	-	0.76
	Site of sampling	Farm	ref	-	-	-	
		Slaughterhouse		-	-	-	0.85
Tigecycline	Type of rearing	Conventional	ref	-	-	-	
		Organic		3.59	1.3	9.93	0.01
		AF		-	-	-	0.13
	Site of sampling	Farm	ref	-	-	-	
		Slaughterhouse		-	-	-	0.15
Colistin	Type of rearing	Conventional ^1^	ref	-	-	-	
		Organic		-	-	-	1.00
		AF		-	-	-	1.00
	Site of sampling	Farm	ref	-	-	-	
		Slaughterhouse		-	-	-	1.00
Meropenem	Type of rearing	Conventional	ref	-	-	-	
		Organic		-	-	-	1.00
		AF		-	-	-	0.98
	Site of sampling	Farm	ref	-	-	-	
		Slaughterhouse		-	-	-	0.61
Cefotaxime	Type of rearing	Conventional	ref	-	-	-	
		Organic		0.39	0.18	0.83	0.01
		AF		-	-	-	0.09
	Site of sampling	Farm	ref	-	-	-	
		Slaughterhouse		-	-	-	0.55
Ceftaxidime	Type of rearing	Conventional	ref	-	-	-	
		Organic		0.36	0.15	0.83	0.02
		AF		-	-	-	0.44
	Site of sampling	Farm	ref	-	-	-	
		Slaughterhouse		-	-	-	0.85
Ampicillin	Type of rearing	Conventional	ref	-	-	-	
		Organic		-	-	-	0.14
		AF		-	-	-	0.16
	Site of sampling	Farm	ref	-	-	-	
		Slaughterhouse		-	-	-	0.60
Nalidixic acid	Type of rearing	Conventional	ref	-	-	-	
		Organic		-	-	-	
		AF		2.32	1.07	5.07	0.03
	Site of sampling	Farm	ref	-	-	-	
		Slaughterhouse		-	-	-	0.48
Ciprofloxacin	Type of rearing	Conventional	ref	-	-	-	
		Organic		0.46	0.22	0.97	0.04
		AF		-	-	-	0.06
	Site of sampling	Farm	ref	-	-	-	
		Slaughterhouse		-	-	-	0.17
Tetracycline	Type of rearing	Conventional	ref	-	-	-	
		Organic		-	-	-	0.07
		AF		-	-	-	0.23
	Site of sampling	Farm	ref	-	-	-	
		Slaughterhouse		-	-	-	0.45
Azitromycin	Type of rearing	Conventional	ref	-	-	-	
		Organic		4.39	1.86	10.39	0.001
		AF		-	-	-	0.99
	Site of sampling	Farm	ref	-	-	-	
		Slaughterhouse		-	-	-	0.05
Gentamicin	Type of rearing	Conventional	ref	-	-	-	
		Organic		2.34	1.13	4.85	0.02
		AF		-	-	-	0.13
	Site of sampling	Farm	ref	-	-	-	
		Slaughterhouse		-	-	-	0.89
Sulfamethoxazole	Type of rearing	Conventional	ref	-	-	-	
		Organic		-	-	-	0.2
		AF		-	-	-	0.24
	Site of sampling	Farm	ref	-	-	-	
		Slaughterhouse		-	-	-	0.94
Chloramphenicol	Type of rearing	Conventional	ref	-	-	-	
		Organic		0.42	0.2	0.9	0.03
		AF		0.3	0.14	0.64	0.002
	Site of sampling	Farm	ref	-	-	-	
		Slaughterhouse		-	-	-	0.22
Trimethoprim	Type of rearing	Conventional	ref	-	-	-	
		Organic		-	-	-	0.38
		AF		-	-	-	0.85
	Site of sampling	Farm	ref	-	-	-	
		Slaughterhouse		-	-	-	0.36

* Odds ratio. Only the significant values are reported. ^a^ Reference category. *p* ≤ 0.05.

## Data Availability

Not applicable.

## Supplementary Materials

The following are available online at https://www.mdpi.com/article/10.3390/antibiotics10111321/s1, Table S1. Distribution of minimal inhibitory concentration (MIC) of *E. coli* strains isolated from organic, antibiotic-free and conventional systems toward the selected antimicrobials. Table S2: Resistance patterns in *E. coli* isolated from antibiotic-free (AF), organic (O), and conventional (C) samples.

## References

[1] Ilea R.C. (2008). Intensive Livestock Farming: Global Trends, Increased Environmental Concerns, and Ethical Solutions. J. Agric. Environ. Ethic..

[2] Alonso C.A., Zarazaga M., Ben Sallem R., Jouini A., BEN Slama K., Torres C. (2017). Antibiotic resistance inEscherichia coliin husbandry animals: The African perspective. Lett. Appl. Microbiol..

[3] Chimera E.T., Fosgate G.T., Etter E.M., Boulangé A., Vorster I., Neves L. (2021). A one health investigation of pathogenic trypanosomes of cattle in Malawi. Prev. Veter. Med..

[4] Castanon J. (2007). History of the Use of Antibiotic as Growth Promoters in European Poultry Feeds. Poult. Sci..

[5] (2017). Drug-Resistant Infections: A Threat to Our Economic Future.

[6] Roila R., Ranucci D., Valiani A., Galarini R., Servili M., Branciari R. (2019). Antimicrobial and anti-biofilm activity of olive oil by-products against Campylobacter spp. isolated from chicken meat. Acta Sci. Pol. Technol. Aliment..

[7] Grace D. (2015). Review of evidence on antimicrobial resistance and animal agriculture in developing countries. Evid. Demand Int. Livest. Res. Inst..

[8] Young A.L., Nicol M.P., Moodley C., Bamford C.M. (2019). The accuracy of extended-spectrum beta-lactamase detection in Escherichia coli and Klebsiella pneumoniae in South African laboratories using the Vitek 2 Gram-negative susceptibility card AST-N255. South. Afr. J. Infect. Dis..

[9] Agyare C., Boamah V.E., Zumbi C.N., Osei F.B. (2018). Antibiotic Use in Poultry Production and Its Effects on Bacterial Resistance. Antimicrob Resist. A Glob. Threat..

[10] Li J., Fu Y., Zhang J., Wang Y., Zhao Y., Fan X., Yu L., Wang Y., Zhang X., Li C. (2019). Efficacy of tigecycline monotherapy versus combination therapy with other antimicrobials against carbapenem-resistant Acinetobacter baumannii sequence type 2 in Heilongjiang Province. Ann. Palliat. Med..

[11] Giamarellou H. (2016). Epidemiology of infections caused by polymyxin-resistant pathogens. Int. J. Antimicrob. Agents.

[12] Poirel L., Jayol A., Nordmann P. (2017). Polymyxins: Antibacterial Activity, Susceptibility Testing, and Resistance Mechanisms Encoded by Plasmids or Chromosomes. Clin. Microbiol. Rev..

[13] He T., Wang R., Liu D., Walsh T., Zhang R., Lv Y., Ke Y., Ji Q., Wei R., Liu Z. (2019). Emergence of plasmid-mediated high-level tigecycline resistance genes in animals and humans. Nat. Microbiol..

[14] Sun J., Chen C., Cui C.-Y., Zhang Y., Liu X., Cui Z.-H., Ma X.-Y., Feng Y.-J., Fang L.-X., Lian X.-L. (2019). Plasmid-encoded tet(X) genes that confer high-level tigecycline resistance in Escherichia coli. Nat. Microbiol..

[15] Skov R.L., Monnet D.L. (2016). Plasmid-mediated colistin resistance (mcr-1 gene): Three months later, the story unfolds. Eurosurveillance.

[16] Schwarz S., Johnson A.P. (2016). Transferable resistance to colistin: A new but old threat: Table 1. J. Antimicrob. Chemother..

[17] Countries should Reduce Use of Colistin in Animals to Decrease the Risk of Antimicrobial Resistance|European Medicines Agency. https://www.ema.europa.eu/en/news/countries-should-reduce-use-colistin-animals-decrease-risk-antimicrobial-resistance.

[18] Rhouma M., Beaudry F., Letellier A. (2016). Resistance to colistin: What is the fate for this antibiotic in pig production?. Int. J. Antimicrob. Agents.

[19] Kempf I., Jouy E., Chauvin C. (2016). Colistin use and colistin resistance in bacteria from animals. Int. J. Antimicrob. Agents.

[20] Jeannot K., Bolard A., Plésiat P. (2017). Resistance to polymyxins in Gram-negative organisms. Int. J. Antimicrob. Agents.

[21] Webb H.E., Angulo F.J., Granier S.A., Scott H.M., Loneragan G.H. (2017). Illustrative examples of probable transfer of resistance determinants from food animals to humans: Streptothricins, glycopeptides, and colistin. F1000Research.

[22] Webster P. (2009). The perils of poultry. Can. Med. Assoc. J..

[23] Dutil L., Irwin R., Finley R., Ng L.K., Avery B., Boerlin P., Bourgault A.-M., Cole L., Daignault D., Desruisseau A. (2010). Ceftiofur Resistance inSalmonella entericaSerovar Heidelberg from Chicken Meat and Humans, Canada. Emerg. Infect. Dis..

[24] Antimicrobial Resistance|FDA. https://www.fda.gov/animal-veterinary/safety-health/antimicrobial-resistance.

[25] Dierikx C.M., Van Der Goot J.A., Smith H.E., Kant A., Mevius D.J. (2013). Presence of ESBL/AmpC -Producing Escherichia coli in the Broiler Production Pyramid: A Descriptive Study. PLoS ONE.

[26] Marangoni F., Corsello G., Cricelli C., Ferrara N., Ghiselli A., Lucchin L., Poli A. (2015). Role of poultry meat in a balanced diet aimed at maintaining health and wellbeing: An Italian consensus document. Food Nutr. Res..

[27] Perez F., Endimiani A., Hujer K.M., Bonomo R.A. (2007). The continuing challenge of ESBLs. Curr. Opin. Pharmacol..

[28] Black S.R., Weaver K.N., Weinstein R.A., Hayden M.K., Lin M.Y., Lavin M.A., Gerber S.I. (2015). Regional Infection Control Assessment of Antibiotic Resistance Knowledge and Practice. Infect. Control. Hosp. Epidemiol..

[29] Pesciaroli M., Magistrali C.F., Filippini G., Epifanio E.M., Lovito C., Marchi L., Maresca C., Massacci F.R., Orsini S., Scoccia E. (2019). Antibiotic-resistant commensal Escherichia coli are less frequently isolated from poultry raised using non-conventional management systems than from conventional broiler. Int. J. Food Microbiol..

[30] EUCAST: New S, I and R definitions. https://www.eucast.org/newsiandr/.

[31] Shang Y., Kumar S., Oakley B., Kim W.K. (2018). Chicken Gut Microbiota: Importance and Detection Technology. Front. Veter. Sci..

[32] Zhanel G.G., Homenuik K., Nichol K., Noreddin A., Vercaigne L., Embil J., Gin A., Karlowsky J.A., Hoban D.J. (2004). The Glycylcyclines. Drugs.

[33] Rose W.E., Rybak M.J. (2006). Tigecycline: First of a New Class of Antimicrobial Agents. Pharmacother. J. Hum. Pharmacol. Drug Ther..

[34] Sun H., Wan Y., Du P., Liu D., Li R., Zhang P., Wu Y., Fanning S., Wang Y., Bai L. (2020). Investigation of tigecycline resistant Escherichia coli from raw meat reveals potential transmission among food-producing animals. Food Control..

[35] Huang X., Yu L., Chen X., Zhi C., Yao X., Liu Y., Wu S., Guo Z., Yi L., Zeng Z. (2017). High Prevalence of Colistin Resistance and mcr-1 Gene in Escherichia coli Isolated from Food Animals in China. Front. Microbiol..

[36] Ahmed S., Das T., Islam Z., Herrero-Fresno A., Biswas P.K., Olsen J.E. (2020). High prevalence of mcr-1-encoded colistin resistance in commensal Escherichia coli from broiler chicken in Bangladesh. Sci. Rep..

[37] Majewski M., Łukomska A., Wilczyński J., Wystalska D., Racewicz P., Nowacka-Woszuk J., Pszczola M., Anusz K. (2020). Colistin resistance of non-pathogenic strains of Escherichia coli occurring as natural intestinal flora in broiler chickens treated and not treated with colistin sulphate. J. Veter. Res..

[38] Botsoglou N. (2001). Drug Residues in Foods: Pharmacology, Food Safety, and Analysis.

[39] Goetting V., Lee K.A., Tell L.A. (2011). Pharmacokinetics of veterinary drugs in laying hens and residues in eggs: A review of the literature. J. Veter. Pharmacol. Ther..

[40] Lim L.M., Ly N., Anderson D., Yang J.C., Macander L., Jarkowski A., Forrest A., Bulitta J., Tsuji B.T. (2010). Resurgence of Colistin: A Review of Resistance, Toxicity, Pharmacodynamics, and Dosing. Pharmacother. J. Hum. Pharmacol. Drug Ther..

[41] Plaza-Rodríguez C., Alt K., Grobbel M., Hammerl J.A., Irrgang A., Szabo I., Stingl K., Schuh E., Wiehle L., Pfefferkorn B. (2021). Wildlife as Sentinels of Antimicrobial Resistance in Germany?. Front. Veter. Sci..

[42] Swift B.M., Bennett M., Waller K., Dodd C., Murray A., Gomes R.L., Humphreys B., Hobman J.L., Jones M.A., Whitlock S.E. (2018). Anthropogenic environmental drivers of antimicrobial resistance in wildlife. Sci. Total. Environ..

[43] Ibekwe A.M., Murinda S.E., Debroy C., Reddy G.B. (2016). Potential pathogens, antimicrobial patterns and genotypic diversity ofEscherichia coliisolates in constructed wetlands treating swine wastewater. FEMS Microbiol. Ecol..

[44] Alhendi A.B., Musa Homeida A.-A., Gaili E.-S., Alhendi A.B., Homeida A.-A.M. (2000). Drug residues in broiler chickens fed with antibiotics in ration GAILI: Drug residues in broiler chickens fed with antibiotics in ration. Vet. Arh..

[45] Zakeri B., Wright G.D. (2008). Chemical biology of tetracycline antibioticsThis paper is one of a selection of papers published in this Special Issue, entitled CSBMCB—Systems and Chemical Biology, and has undergone the Journal's usual peer review process. Biochem. Cell Biol..

[46] Kabir J., Umoh V., Audu-Okoh E., Umoh J., Kwaga J. (2004). Veterinary drug use in poultry farms and determination of antimicrobial drug residues in commercial eggs and slaughtered chicken in Kaduna State, Nigeria. Food Control..

[47] Carattoli A. (2008). Animal reservoirs for extended spectrum β-lactamase producers. Clin. Microbiol. Infect..

[48] Musa L., Proietti P.C., Branciari R., Menchetti L., Bellucci S., Ranucci D., Marenzoni M.L., Franciosini M.P. (2020). Antimicrobial Susceptibility of *Escherichia coli* and ESBL-Producing *Escherichia coli* Diffusion in Conventional, Organic and Antibiotic-Free Meat Chickens at Slaughter. Animals.

[49] Hafez H.M. (2011). Enteric Diseases of Poultry with Special Attention to Clostridium perfringens. Pak. Vet. J..

[50] Cooper K., Songer J.G., Uzal F.A. (2013). Diagnosing clostridial enteric disease in poultry. J. Veter. Diagn. Investig..

[51] The European Union (2006). Ban on antibiotics as growth promoters in animal feed enters into effect. Proceedings of the European Commission. Regulation.

[52] Jiménez-Belenguer A., Doménech E., Villagrá A., Fenollar A., Ferrús M.A. (2016). Antimicrobial resistance of Escherichia coli isolated in newly-hatched chickens and effect of amoxicillin treatment during their growth. Avian Pathol..

[53] Burow E., Grobbel M., Tenhagen B.-A., Simoneit C., Szabó I., Wendt D., Kürbis C., Ladwig-Wiegard M., Banneke S., Käsbohrer A. (2020). Antibiotic Resistance in Escherichia coli from Broiler Chickens After Amoxicillin Treatment in an Experimental Environment. Microb. Drug Resist..

[54] Dohmen W., VAN Gompel L., Schmitt H., Liakopoulos A., Heres L., Urlings B.A., Mevius D., Bonten M.J.M., Heederik D.J.J. (2017). ESBL carriage in pig slaughterhouse workers is associated with occupational exposure. Epidemiol. Infect..

[55] Cui S., Ge B., Zheng J., Meng J. (2005). Prevalence and Antimicrobial Resistance of Campylobacter spp. and Salmonella Serovars in Organic Chickens from Maryland Retail Stores. Appl. Environ. Microbiol..

[56] Miranda J.M., Vázquez B.I., Fente C.A., Calo-Mata P., Cepeda A., Franco C.M. (2008). Comparison of Antimicrobial Resistance in Escherichia coli, Staphylococcus aureus, and Listeria monocytogenes Strains Isolated from Organic and Conventional Poultry Meat. J. Food Prot..

[57] Millman J.M., Waits K., Grande H., Marks A.R., Marks J.C., Price L.B., Hungate B.A. (2013). Prevalence of antibiotic-resistant E. coli in retail chicken: Comparing conventional, organic, kosher, and raised without antibiotics. F1000Research.

[58] Mollenkopf D.F., Cenera J.K., Bryant E.M., King C.A., Kashoma I., Kumar A., Funk J.A., Rajashekara G., Wittum T.E. (2014). Organic or Antibiotic-Free Labeling Does Not Impact the Recovery of Enteric Pathogens and Antimicrobial-ResistantEscherichia colifrom Fresh Retail Chicken. Foodborne Pathog. Dis..

[59] ISO ISO 16649-1:2018—Microbiology of the food chain—Horizontal method for the enumeration of beta-glucuronidase-positive Escherichia coli—Part 1: Colony-count technique at 44 degrees C using membranes and 5-bromo-4-chloro-3-indolyl beta-D-glucuronide. https://www.iso.org/standard/64951.html.

[60] European Committe on Antimicrobial Susceptability Testing Clinical Breakpoints and Dosing of Antibiotics. https://eucast.org/clinical_breakpoints/.

[61] Weinstein M.P., Patel J.B., Bobenchik A.M., Campeau S., Cullen S.K., Galas M.F., Gold H., Humphries R.M., Kirn T.J., Lewis Ii J.S. (2020). M100 Performance Standards for Antimicrobial Susceptibility Testing A CLSI supplement for global application. Performance Standards for Antimicrobial Susceptibility Testing Performance Standards for Antimicrobial Susceptibility Testing.

[62] Bonnedahl J., Järhult J.D. (2014). Antibiotic resistance in wild birds. Upsala J. Med. Sci..

[63] Boiocchi F., Davies M.P., Hilton A.C. (2019). An Examination of Flying Insects in Seven Hospitals in the United Kingdom and Carriage of Bacteria by True Flies (Diptera: Calliphoridae, Dolichopodidae, Fanniidae, Muscidae, Phoridae, Psychodidae, Sphaeroceridae). J. Med. Èntomol..

[64] Bouki C., Venieri D., Diamadopoulos E. (2013). Detection and fate of antibiotic resistant bacteria in wastewater treatment plants: A review. Ecotoxicol. Environ. Saf..

