# Supplementary material for: Susceptibility of Commensal E. coli Isolated from Conventional, Antibiotic-Free, and Organic Meat Chickens on Farms and at Slaughter toward Antimicrobials with Public Health Relevance
Source: Antibiotics (Basel). 2021 Oct 29;10(11):1321. doi: 10.3390/antibiotics10111321 (PMC8614865; doi:10.3390/antibiotics10111321)
Supplement: Supplementary file 1 [file antibiotics-10-01321-s001.zip › antibiotics-1410974-supplementary.pdf]

Table S1. Distribution of minimal inhibitory concentration (MIC) of *E. coli* strains isolated from organic, antibiotic-free and conventional systems toward the selected antimicrobials.

	**Organic**		**Antibiotic-FREE**		**Conventional**	
**Antimicrobials**	MIC values		MIC values		MIC values	
**Tigecycline**	1-4 μg/mL<br>29.3%	≥ 8 μg/mL<br>0%	1-4 μg/mL<br>20.7%	≥ 8 μg/mL<br>0%	1-4 μg/mL<br>10.3%	≥ 8 μg/mL<br>0%
**Colistin**	>2 μg/mL<br>0%		>2 μg/mL<br>0%		>2 μg/mL<br>0%	
**Meropenem**	≥16 μg/mL<br>0%		≥16 μg/mL<br>0%		≥16 μg/mL<br>0%	
**Cefotaxime**	≥4 μg/mL<br>29.3%		≥4 μg/mL<br>36.2%		≥4 μg/mL<br>51.7%	
**Ceftazidime**	≥8 μg/mL<br>8.6%		≥8 μg/mL<br>10.34%		≥8 μg/mL<br>31.%	
**Ampicillin**	16-32 μg/mL<br>0%	≥ 64 μg/mL<br>72.4%	16-32 μg/mL<br>0%	≥ 64 μg/mL<br>70.7%	16-32 μg/mL<br>0%	≥ 64 μg/mL<br>84.5%
**Nalidixic Acid***	32-64 μg/mL<br>6.9%	≥ 128 μg/mL<br>39.7%	32-64 μg/mL<br>0	≥ 128 μg/mL<br>58.6%	32-64 μg/mL<br>0%	≥ 128 μg/mL<br>56.9%
**Ciprofloxacin**	1-4 μg/mL<br>20.7%	≥ 8 μg/mL<br>19.0%	1-4 μg/mL<br>19.0%	≥ 8 μg/mL<br>22.40%	1-4 μg/mL<br>25.9%	≥ 8 μg/mL<br>32.7%
**Tetracycline***	16-32 μg/mL<br>36.20%	≥ 64 μg/mL<br>50.00%	16-32 μg/mL<br>29.30%	≥ 64 μg/mL<br>51.70%	16-32 μg/mL<br>34.50%	≥ 64 μg/mL<br>43.10%
**Azithromycin***	32-64 μg/mL<br>8.60%	> 64 μg/mL<br>20.7%	32-64 μg/mL<br>3.40%	> 64 μg/mL<br>6.9%	32-64 μg/mL<br>6.9%	> 64 μg/mL<br>0%
**Gentamicin**	4-16 μg/mL<br>19%	≥ 32 μg/mL<br>13.8%	4-16 μg/mL<br>13.8%	≥ 32 μg/mL<br>8.6%	4-16 μg/mL<br>5.2%	≥ 32 μg/mL<br>6.9%
**Sulfamethoxazole***	512 μg/mL<br>5.2%	≥ 1024 μg/mL<br>77.6%	512 μg/mL<br>0%	≥ 1024 μg/mL<br>81%	512 μg/mL<br>9%	≥ 1024 μg/mL<br>63.4%
**Chloramphenicol**	16-64 μg/mL<br>24.2%	≥ 128 μg/mL<br>22.4%	16-64 μg/mL<br>6.9%	≥ 128 μg/mL<br>31.%	16-64 μg/mL<br>44.8%	≥ 128 μg/mL<br>22.4%
**Trimethoprim**	8-16 μg/mL<br>17.2%	≥ 32 μg/mL<br>50%	8-16 μg/mL<br>6.9%	≥ 32 μg/mL<br>55.2%	8-16 μg/mL<br>5%	≥ 32 μg/mL<br>58.8%

* based on the CLSI clinical breakpoint.

Table S2. Resistance patterns in *E. coli* isolated from antibiotic-free (AF) organic (O), and conventional (C) samples.

	System												
	AF		O		C		Antimicrobial Resistance Pattern	AF		O		C	
Pattern	*E. coli* (No)	%	*E. coli* (No)	%	*E. coli* (No)	%		E. coli (No)	%	*E.coli* (No)	%	*E.coli* (No)	%
							BL/QUIN	2	3.40%	0	0.00%	0	0.00%
2	3	5.10%	1	1.70%	1	1.70%	TGC/CHL	0	0.00%	1	1.70%	0	0.00%
							TET/AZI	1	1.70%	0	0.00%	0	0.00%
							BL/CHL	0	0.00%	0	0.00%	1	1.70%
							BL/CN/TMP	0	0.00%	1	1.70%	0	0.00%
							BL/QUIN/TMP	0	0.00%	0	0.00%	1	1.70%
							BL/QUIN/TET	0	0.00%	0	0.00%	3	5.10%
							BL/QUIN/SMX	0	0.00%	0	0.00%	1	1.70%
							BL/SMX/TMP	1	1.70%	0	0.00%	0	0.00%
							BL/SMX/CHL	1	1.70%	0	0.00%	1	1.70%
3	8	13.60%	4	6.80%	8	13.60%	TGC/BL/TET	1	1.70%	0	0.00%	0	0.00%
							TGC/QUIN/TET	1	1.70%	0	0.00%	0	0.00%
							SMX/CHL/TMP	1	1.70%	0	0.00%	1	1.70%
							TGC/SMX/CHL	0	0.00%	0	0.00%	1	1.70%
							TET/AZI/SMX	1	1.70%	0	0.00%	0	0.00%
							TET/AZI/TMP	1	1.70%	0	0.00%	0	0.00%
							TET/SMX/TMP	0	0.00%	3	5.10%	0	0.00%
							TET/SMX/CHL	1	1.70%	0	0.00%	0	0.00%
							BL/QUIN/CN/SMX	1	1.70%	0	0.00%	0	0.00%
							CN/SMX/CHL/TMP	1	1.70%	0	0.00%	0	0.00%
							BL/QUIN/TET/TMP	0	0.00%	1	1.70%	1	1.70%
							BL/QUIN/SMX/TMP	0	0.00%	1	1.70%	0	0.00%
							BL/QUIN/TET/SMX	2	3.40%	1	1.70%	3	5.10%
							BL/SMX/CHL/TMP	0	0.00%	2	3.40%	1	1.70%

							BL/TET/SMX/CHL	1	1.70%	0	0.00%	1	1.70%
4	10	17.00%	12	20.40%	7	13.60%	BL/TET/SMX/TMP	3	5.10%	1	1.70%	2	3.40%
							QUIN/TET/CN/SMX	0	0.00%	1	1.70%	0	0.00%
							QUIN/TET/SMX/CHL	0	0.00%	1	1.70%	0	0.00%
							QUIN/TET/SMX/TMP	1	1.70%	0	0.00%	0	0.00%
							TGC/BL/TET/SMX	0	0.00%	1	1.70%	0	0.00%
							TGC/BL/CN/SMX	0	0.00%	1	1.70%	0	0.00%
							TGC/TET/AZI/TMP	0	0.00%	1	1.70%	0	0.00%
							TET/AZI/SMX/TMP	0	0.00%	1	1.70%	0	0.00%
							TGC/TET/SMX/TMP	1	1.70%	0	0.00%	0	0.00%
							BL/QUIN/CN/SMX/TMP	0	0.00%	1	1.70%	0	0.00%
							CN/QUIN/TET/SMX/TMP	0	0.00%	0	0.00%	1	1.70%
							BL/QUIN/TET/CN/CHL	0	0.00%	1	1.70%	0	0.00%
							BL/QUIN/TET/CN/TMP	1	1.70%	0	0.00%	0	0.00%
							BL/QUIN/SMX/CHL/TMP	0	0.00%	0	0.00%	1	1.70%
							BL/QUIN/TET/CN/SMX	1	1.70%	1	1.70%	0	0.00%
							BL/QUIN/TET/CHL/TMP	0	0.00%	0	0.00%	4	6.80%
							BL/QUIN/TET/SMX/CHL	1	1.70%	1	1.70%	4	6.80%
							BL/QUIN/TET/SMX/TMP	6	10.20%	3	5.10%	1	1.70%
							BL/TET/CN/SMX/CHL	0	0.00%	1	1.70%	0	0.00%
5	13	22.10%	18	30.60%	17	28.90%	BL/TET/CN/SMX/TMP	1	1.70%	0	0.00%	0	0.00%
							BL/TET/SMX/CHL/TMP	1	1.70%	3	5.10%	4	6.80%
							BL/TET/AZI/CHL/TMP	0	0.00%	0	0.00%	1	1.70%
							TGC/BL/QUIN/TET/AZI	0	0.00%	2	3.40%	0	0.00%
							TGC/BL/QUIN/TET/TMP	0	0.00%	0	0.00%	1	1.70%
							TGC/BL/QUIN/TET/SMX	1	1.70%	0	0.00%	0	0.00%
							TGC/BL/TET/AZI/CHL	0	0.00%	1	1.70%	0	0.00%
							TGC/BL/TET/CN/TMP	0	0.00%	1	1.70%	0	0.00%
							TGC/TET/CN/SMX/TMP	0	0.00%	1	1.70%	0	0.00%
							TGC/BL/TET/SMX/TMP	1	1.70%	0	0.00%	0	0.00%

							TGC/TET/AZI/SMX/TMP	0	0.00%	1	1.70%	0	0.00%
							TET/AZI/SMX/CHL/TMP	0	0.00%	1	1.70%	0	0.00%
							BL/QUIN/TET/CN/SMX/CHL	0	0.00%	0	0.00%	1	1.70%
							BL/QUIN/TET/CN/SMX/TMP	3	5.10%	2	3.40%	0	0.00%
							BL/QUIN/TET/AZI/CN/SMX	0	0.00%	2	3.40%	0	0.00%
							BL/QUIN/TET/AZI/SMX/CHL	0	0.00%	1	1.70%	0	0.00%
							BL/QUIN/TET/AZI/SMX/CHL	1	1.70%	0	0.00%	0	0.00%
							BL/QUIN/TET/SMX/CHL/TMP	7	11.90%	5	8.50%	8	13.60%
6	14	23.80%	14	23.80%	10	17.00%	BL/QUIN/TET/AZI/SMX/TMP	0	0.00%	1	1.70%	0	0.00%
							TGC/BL/QUIN/TET/SMX/CHL	1	1.70%	0	0.00%	1	1.70%
							BL/QUIN/TET/AZI/SMX/TMP	0	0.00%	1	1.70%	0	0.00%
							TGC/BL/TET/AZI/SMX/CHL	0	0.00%	1	1.70%	0	0.00%
							TGC/BL/QUIN/TET/SMX/TMP	1	1.70%	0	0.00%	0	0.00%
							TGC/QUIN/TET/AZI/SMX/CHL	0	0.00%	1	1.70%	0	0.00%
							TGC/TET/AZI/SMX/CHL/TMP	1	1.70%	0	0.00%	0	0.00%
							TGC/BL/QUIN/TET/CN/SMX/CHL	0	0.00%	1	1.70%	0	0.00%
							BL/QUIN/TET/CN/SMX/CHL/TMP	2	3.40%	3	5.20%	7	12.10%
7	5	8.70%	6	10.20%	8	13.60%	TGC/BL/QUIN/TET/AZI/SMX/CHL	1	1.70%	0	0.00%	0	0.00%
							TGC/BL/QUIN/TET/SMX/CHL/TMP	2	3.40%	1	1.70%	1	1.70%
							TGC/QUIN/TET/AZI/CN/SMX/TMP	0	0.00%	1	1.70%	0	0.00%
							BL/QUIN/TET/AZI/CN/SMX/CHL/TMP	0	0.00%	1	1.70%	0	0.00%
8	1	1.70%	2	3.40%	1	1.70%	TGC/BL/QUIN/TET/CN/SMX/CHL/TMP	0	0.00%	1	1.70%	0	0.00%
							TGC/BL/QUIN/TET/AZI/CN/SMX/TMP	1	1.70%	0	0.00%	0	0.00%
							TGC/BL/QUIN/TET/AZI/SMX/CHL/TMP	0	0.00%	0	0.00%	1	1.70%

BL = beta-lactams (MERO, FOT, TAZ, AMP); QUIN = quinolones (CIP, NA), SMX = sulfamethoxazole, TET = tetracycline, CN = gentamicin, TGC = tigecycline, AZI = azitromycin, CHL = chloramohenicol, TMP = trimethoprim.
